# Far-infrared radiation protects viability in a cell model of Spinocerebellar Ataxia by preventing polyQ protein accumulation and improving mitochondrial function

**DOI:** 10.1038/srep30436

**Published:** 2016-07-29

**Authors:** Jui-Chih Chang, Shey-Lin Wu, Fredrik Hoel, Yu-Shan Cheng, Ko-Hung Liu, Mingli Hsieh, August Hoel, Karl Johan Tronstad, Kuo-Chia Yan, Ching-Liang Hsieh, Wei-Yong Lin, Shou-Jen Kuo, Shih-Li Su, Chin-San Liu

**Affiliations:** 1 Vascular and Genomic Center, Changhua Christian Hospital, Changhua 50094, Taiwan; 2 Department of Neurology, Changhua Christian Hospital, Changhua 50094, Taiwan; 3 Department of Biomedicine, University of Bergen, 5020 Bergen, Norway; 4 Department of Life Science, Tunghai University, Taichung 40704, Taiwan; 5 Department of Dermatology, Changhua Christian Hospital, Changhua 50094, Taiwan; 6 Department of Chinese Medicine, Obstetrics and Gynecology, Dermatology, and Urology, China Medical University Hospital, Taichung 40447, Taiwan; 7 School of Chinese Medicine, Graduate Institute of Integrated Medicine, Research Center for Chinese Medicine and Acupuncture, China Medical University, Taichung 40447, Taiwan; 8 Departments of Medical Research, Obstetrics and Gynecology, Dermatology, and Urology, China Medical University Hospital, Taichung 40447, Taiwan; 9 Department of Surgery, Changhua Christian Hospital, Changhua 50094, Taiwan; 10 Division of Endocrinology and Metabolism, Department of Internal Medicine, Changhua Christian Hospital, Changhua 50094, Taiwan; 11 Institute of Medicine, Chung Shan Medical University, Taichung 40201, Taiwan

## Abstract

Far infrared radiation (FIR) is currently investigated as a potential therapeutic strategy in various diseases though the mechanism is unknown. Presently, we tested if FIR mediates beneficial effects in a cell model of the neurodegenerative disease spinocerebellar ataxia type 3 (SCA3). SCA3 is caused by a mutation leading to an abnormal polyglutamine expansion (PolyQ) in ataxin-3 protein. The consequent aggregation of mutant ataxin-3 results in disruption of vital cell functions. In this study, neuroblastoma cells (SK-N-SH) was transduced to express either non-pathogenic ataxin-3-26Q or pathogenic ataxin-3-78Q proteins. The cells expressing ataxin-3-78Q demonstrated decreased viability, and increased sensitivity to metabolic stress in the presence rotenone, an inhibitor of mitochondrial respiration. FIR exposure was found to protect against these effects. Moreover, FIR improved mitochondrial respiratory function, which was significantly compromised in ataxin-3-78Q and ataxin-3-26Q expressing cells. This was accompanied by decreased levels of mitochondrial fragmentation in FIR treated cells, as observed by fluorescence microscopy and protein expression analysis. Finally, the expression profile LC3-II, Beclin-1 and p62 suggested that FIR prevent the autophagy inhibiting effects observed in ataxin-3-78Q expressing cells. In summary, our results suggest that FIR have rescuing effects in cells expressing mutated pathogenic ataxin-3, through recovery of mitochondrial function and autophagy.

Neurodegenerative PolyQ diseases are a group of relatively rare dominantly inherited disorders that are characterized by progressive and selective loss of neuronal cell bodies, dendrites and/or axons in the central nervous system. They are caused by poly-glutamine expansion (poly-glutamine-tract; PolyQ) in the expressed protein of the mutated gene. Spinocerebellar ataxia type 3 (SCA3), Huntington’s disease (HD) and spinal bulbar muscular atrophy (SBMA) are well known PolyQ diseases^1^,^2^. Currently, there is no effective treatment for these diseases, and more research is therefore required to improve the outcome for these patients. In the present work we studied the cell protective effects of far infrared radiation (FIR), as a strategy to prevent the damaging effects of PolyQ proteins in cultured cells.

In SCA3 (also known as Machado-Joseph disease or MJD), the ataxin-3 gene is mutated and typically contains an extension with 60-87 CAG-repeats. The disease often presents between age 45 and 70, depending on the number of CAG-repeats. SCA3 is characterized by an enlargement of the fourth ventricle due to degeneration of the brainstem and cerebellum and progressively develops into muscular atrophy with ataxia^3^. In common with the other neurodegenerative PolyQ diseases, the pathological mechanism of SCA3 involves aggregation of the mutated protein, mitochondrial dysfunction, cellular stress and ultimately cell death^4^.

Mitochondria are organelles that play crucial roles in maintenance of cellular homeostasis and there is a clear link between mitochondrial dysfunction and neurodegenerative diseases^5^. These organelles contribute a major part of cellular ATP via oxidative phosphorylation, which involves the electron transport chain (ETC) of protein complexes in the inner mitochondrial membrane. Any disturbance in this machinery typically leads to energy deficiency and/or production of reactive oxygen species (ROS), and thereby cellular stress and cell death^4^. Such mechanisms have for instance been shown in Parkinson’s disease (PD), where the pathology is tightly linked to ETC complex I dysfunction and protein aggregation^6^. Impaired ETC function has also been associated with SCA3 and other neurodegenerative disorders^7^,^8^. One mechanism that seems to play an important role to protect against harmful effects of ETC dysfunction is autophagy. Autophagy, which is commonly induced by cellular stress, serves to support cell survival by facilitating removal of damaged cell components^9^. Central regulators of autophagy that have been linked to SCA3 and other neurodegenerative disorders include P62^10^ and Beclin-1^11^, which participate in the coordination of autophagosomes where cellular components are degraded.

Changes in mitochondrial morphology often occur in parallel with adjustments in energy metabolism and responses to stress^12^. This phenomenon is termed mitochondrial dynamics, and is regulated by proteins such as optic atrophy 1 (OPA1), mitofusin 2 (MFN2) and Dynamin-related protein 1 (Drp1), which coordinate events of mitochondrial fission and fusion. Cellular stress, autophagy and cell death are commonly associated with mitochondrial fragmentation, as also observed in neurodegenerative disorders^13^,^14^. The mechanisms of mitochondrial dynamics in neurodegenerative disorders are not completely understood, but are likely to involve common stress responses as well as specific interactions between mutated proteins and regulators of mitochondrial morphology^15^.

FIR have previously been reported to mediate therapeutic effects *in vitro* and *in vivo* on vascular endothelium^16^,^17^ and damaged nerves in rats^18^ but the potential therapeutic effects of FIR in SCA3 still unknown. FIR therapy utilizes longer wavelengths of the infrared spectrum than the established near infrared-radiation therapy (NIR) and regulatory mechanisms have been showed to involve the photoreactive complexes of the ETC^19^. Thus, the purpose of this study was to investigate effects of FIR and implicating mitochondrial role in human neural SK-N-SH cells expressing mutated ataxin-3 with 78 glutamine residues, which known to cause SCA3 pathology.

## Results

### Cell protective effects of FIR in cells expressing pathogenic ataxin-3-78Q

**Figure 1:**
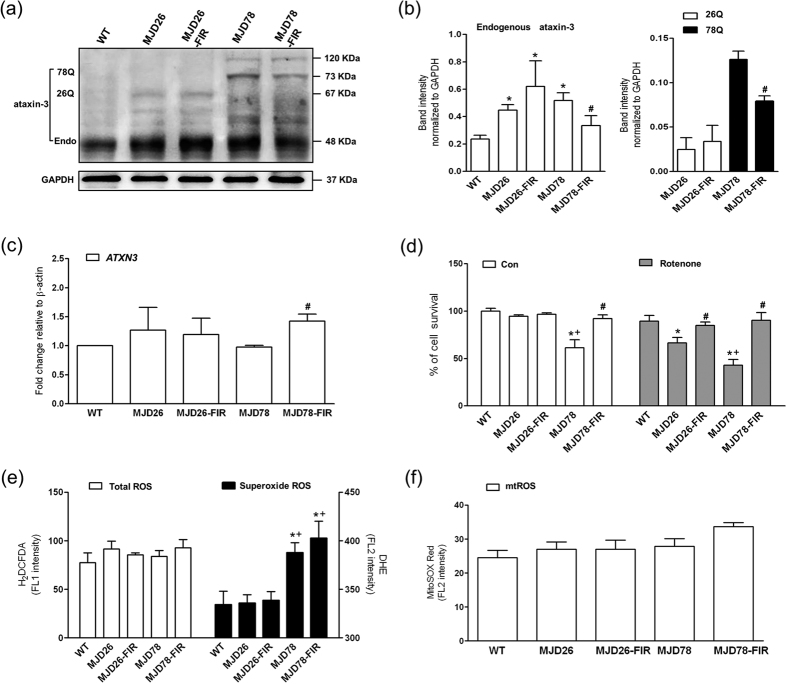
Expression of ataxin-3 with non-pathologic and pathologic polyQ, cell viability and oxidative stress in MJD cells after 3-day FIR treatment. (**a**) Western blot analyzed the protein expressions of ataxin-3 including (**b**) the endogenous ataxin-3 and the polyQ expansion of 26 (26Q) or 78 CAG-repeat expansion (78Q) were quantified in human neuroblastoma cells (SK-N-SH) overexpressing 26- (26Q, internal control, MJD26) and 78-CAG repeats (MJD78) in *ATXN3* gene with or with FIR treatment. The protein quantification was calculated by three-time independent analysis at least. (**c**) The mRNA level of *ATXN3* was detected by using the RT-PCR with the primers flanking non-GAC repeat regions. (**d**) Cell viability and sensitivity of rotenone (100 nM)-induced cell death was evaluated by PI staining using a flow cytometry. The oxidative stress was comprehensively assessed by flow cytometry analysis of (**e**) intracellular (Total) ROS (H_2_DCFDA staining), superoxide (DHE staining) and (**f**) mitochondrial superoxide (mtROS, MitoSOX Red staining). *p < 0.05, compare to WT group; ^#^*p* < 0.05, compare to each non-treated group of MJD cells; ^+^*p* < 0.05, compare to non-treated MJD26 group.

In order to study the effects of FIR on SCA3 pathology we used the SK-N-SH cell line, a neuroblastoma cell line that has a neuronal pre-cursor phenotype and is commonly used to model neurodegenerative diseases. In these cells, we inserted expression vectors for either green fluorescence protein (GFP)-tagged full-length *ATXN3* with 26 (ataxin-3-26Q-GFP, internal control) or 78 glutamine residues (ataxin-3-78Q-GFP). These cells were referred to as MJD26 and MJD78, respectively. Expressions of the respective PolyQ proteins with a mass of 67 KDa and 73 KDa in the MJD26 and MJD78 cells were confirmed by western blotting (Fig. 1a,b). Both of MJD26 and MJD78 expressed dramatic increase of endogenous ataxin-3 compared to the wild type (WT) because the late-passaged passage numbers of transgenic cells at 15–25 were used in the present study (see Supplementary Information Figure S1). FIR exposure decreased both endogenous and mutated ataxin-3 protein levels in contrary to the mRNA level of *ATXN3* (Fig. 1c) in the MJD78 cells, whereas no significant effects were observed in the MJD26 cells (Fig. 1a,b). Moreover, FIR exposure did not affect the performance of 120-kDa ataxin-3 protein aggregate in MJD78 cells (Fig. 1a).

In parallel experiments, expression of ataxin-3-78Q-GFP, but not ataxin-3-26Q-GFP, was found to reduce cell viability compared to non-modified cells (Fig. 1d). FIR exposure had a rescuing effect in MJD78 cells, and thereby reestablished viability to the same level as in MJD26 and WT cells. To evaluate the effect of FIR on the cellular capability to tolerate energetic stress, we employed ETC complex I inhibitor rotenone with a moderate concentration of 100 nM, the minimum dose to induce the statistically significant differences in cell survival of MJD cells, to avoid irreversible damage in MJD78 cells. We found that the MJD78 cells had significant decrease of cell viability in presence of 100 nM rotenone compared to WT and MJD26 cells (Fig. 1d). These data also showed that the MJD26 cells were more sensitive to rotenone relative to WT cells. FIR exposure completely restored the viability in MJD26 and MJD78 cells under these conditions, to the same level as in WT cells. To determine if these differences between the cell types could be explained by oxidative stress introduced by the PolyQ protein, we measured cellular ROS formation. We found no change in the total level of ROS, nor in mitochondrial ROS, between the cell lines; however, the MJD78 cells demonstrated a significant increase in superoxide production and FIR did not protect against these effects (Fig. 1e). In contrast, FIR exposure caused a mild increase in the production of superoxide and mitochondrial ROS (Fig. 1e,f). In summary, these data suggest that FIR has cytoprotective effects in MJD78 cells under conditions of cellular stress, via a mechanism that does not seem to involve ROS scavenging.

### Protection of mitochondrial respiration and morphology

**Figure 2:**
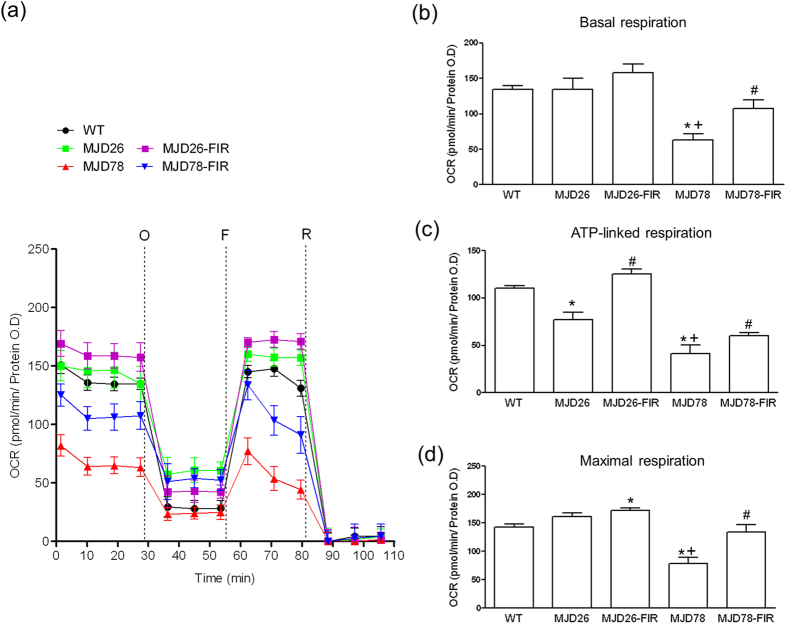
Seahorse XF-24 instruments analysis of oxygen consumption rate (OCR). (**a**) Mitochondrial respiration reflected by OCR levels was detected in wild-type (WT), cell overexpressing 26- (internal control) and 78-CAG repeats (MJD78) in *ATXN3* gene with or with FIR treatment under basal conditions or following the addition of oligomycin (O, 1 μM), the uncoupler FCCP (F, 0.5 μM) or the electron transport inhibitor Rotenone (R, 2 μM) (n  =  5). (**b**) The rates of basal respiration, (**c**) ATP-linked respiration, (**d**) maximal respiratory capacity was quantified by normalization of OCR level to the total protein OD values. *p < 0.05, compare to WT group; ^+^*p* < 0.05, compare to non-treated MJD26 group; ^#^*p* < 0.05, compare to each non-treated group of MJD cells.

To investigate how mitochondrial respiration was affected in our SCA3 cell model, we measured oxygen consumption rates (Fig. 2). The data demonstrated that the basal respiratory rate (Fig. 2b), as well as ATP-linked respiration (Fig. 2c) and respiratory capacity (Fig. 2d) was significantly reduced in the MJD78 cells in contrast to MJD26 cells which only a significantly decrease in ATP-linked respiration (Fig. 2c). FIR treatment not only increased ATP-linked respiration in MJD26 cells but also increased basal and ATP-linked respiratory rates and completely restored respiratory capacity in MJD78 cells compared to each non-treated control cells (Fig. 2b–d).

**Figure 3:**
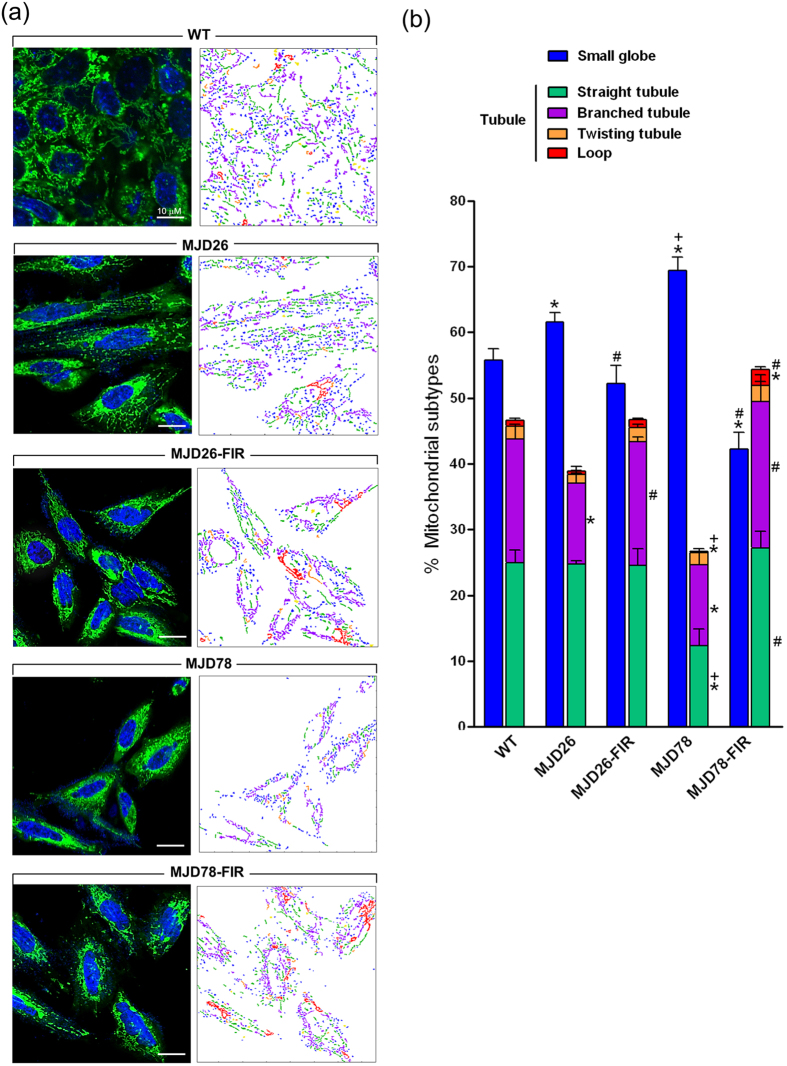
Alteration of mitochondrial morphology in FIR-treated MJD cells. (**a**) Morphology of mitochondria stained by MitoTracker Green probe was observed by a confocal fluorescence microscope in MJD26 and MJD78 cells with or without 3-day FIR treatment. The classification and quantification of mitochondrial morphology were further analyzed by using an automatic system. The color-coded labeling showed the composition of mitochondrial subtypes in each group of representative cells as right-side panel shown in (**a**). The six distinct mitochondrial subtypes were classified as class 2 as follows: small globe (blue) and tubule including straight tubule (green), branched tubule (purple), twisting tubule (orange) and loop (red). The type of swollen globed (yellow) representative of dysfunctional mitochondria was excluded from the qualification of mitochondrial subtypes. (**b**) Mitochondrial subtypes were quantified by calculating the percentage of each mitochondrial subtype in total mitochondrial population. Images from three independent areas contained about 250–350 mitochondrial subjects from about 4–6 cells in each was analyzed at each group. *p < 0.05, compare to WT group; ^+^*p* < 0.05, compare to MJD26 group; ^#^p < 0.05, compare to each non-treated group of MJD cells.

Fluorescence microscopy (Fig. 3a, left panel) and quantitative image analysis^20^ (Fig. 3a, right panel) was performed to investigate if the effects on mitochondrial respiration in MJD cells were associated with mitochondrial fragmentation. The analysis found wherever MJD26 or MJD78 cells had a moderate increase in small globular mitochondria with concomitant decrease tubular mitochondria in MJD26 and MJD78 compared to the WT cells (Fig. 3b). However, mitochondrial fragmentation was more dramatic in MJD78 than in MJD26 cells (Fig. 3b). This effect was, however, not seen in FIR treated cells. There was a significant increase of branched tubules in FIR-treated MJD26 cells as well as straight, branched and loop tubules in FIR-treated MJD 78 cells relative to each non-treated control cells (Fig. 3b).

**Figure 4:**
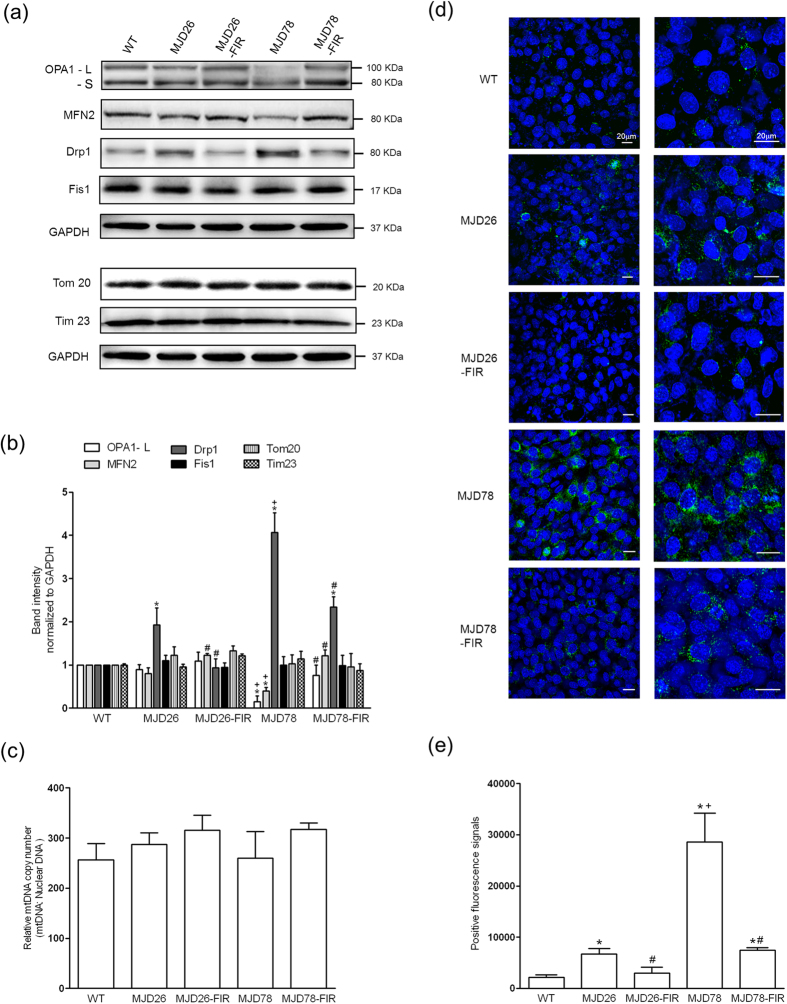
Performance of mitochondrial dynamic-related proteins and biogenesis in SCA3 cellular model under FIR treatment. (**a**) Western blot analyzed the expression of mitochondrial fusion (Optic Atrophy 1, OPA1 and Mitofusin 2, MFN2) and fission proteins (Dynamin-related protein 1, Drp1 and Mitochondrial fission 1 protein, Fis1) as well as mitochondrial marker proteins (Tom 20 and Tim 23). OPA1 has two types of isoforms: long isoforms (OPA1-L) and short isoforms (OPA1-S). Only the L-OPA1 was fusion competent. (**b**) The above-mentioned protein expression was quantified by normalized Glyceraldehyde 3-phosphate dehydrogenase (GAPDH) (n = 3). (**c**) Mitochondrial DNA (mtDNA) copy number per diploid nuclear genome was analyzed by quantitative PCR to confirm the mitochondrial biogenesis (n = 4). (**d**) Expression of mitochondrial fission was further confirmed by examination of phosphorylation of Drp1 (Green color) at Serine 616 (p-Drp1) using immunofluorescent staining. The Hoechst 33342 was applied to the cell-permeant nuclear counterstain (blue color). (**e**) Fluorescent intensity of p-Drp1 was calculated by counting five independent areas at same magnification in each group (n = 3). *p < 0.05, compare to WT group; ^+^*p* < 0.05, compare to MJD26 group; ^#^p < 0.05, compare to each non-treated group of MJD cells.

To further investigate this observation, we measured expression of mitochondrial shaping proteins and amount (Fig. 4). Expression of the mitochondrial fission protein Drp1 was individually found to be almost 1.8-fold and 4.2-fold increases in MJD 26 and MJD78 cells, compared with WT cells. Simultaneously, only MJD78 cells were accompanied with a significant reduction in the mitochondrial fusion proteins OPA1 and MFN2 (Fig. 4a). Interestingly, FIR treatment not only nearly normalized the expression of these proteins in MJD78 but also had effects in MJD26 cells including a significant increase and decreased of MFN2 and Drp1 (Fig. 4a,b). Although Drp1 expression was still relatively high in FIR-treated MJD78 cells relative to WT cells, it was significantly reduced compared to untreated MJD 78 cells. Mitochondrial amount measured by protein analysis of TOM 20 and TOM 23 (Fig. 4a) as well as mitochondrial DNA (Fig. 4c), indicated no significant changes in the amount of mitochondrial biomass. Due to the dramatic reduction of Drp1 in FIR- treated MJD cells, examination of phosphorylated Drp1 protein at Serine 616 measured by immunofluorescent staining was used to confirm the reality of mitochondrial fission (Fig. 4d). The similar performances of phosphorylated Drp-1 (Green fluorescence, Fig. 4d) with Drp1 protein (Fig. 4a,b) were observed in MJD cells with or without FIR treatment. MJD78 cells expressed higher level of phosphorylated Drp-1 relative to MJD26 cells. FIR treatment significantly decreased the extensive expression of phosphorylated Drp-1 in both MJD cells (Fig. 4d,e). Hence, although depression of mitochondrial function and morphology in MJD26 cells was not serious than in MJD78 cells, FIR treatment was found to protect against impairment of mitochondrial respiration, and associated mitochondrial fragmentation and protein expression in both MJD cells.

### Induction of autophagy

**Figure 5:**
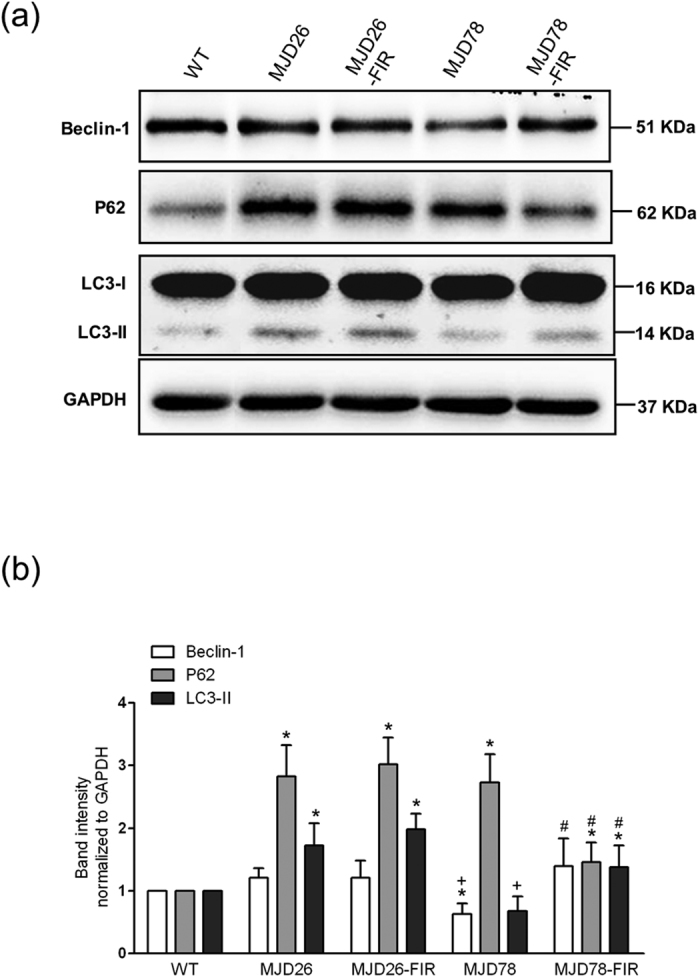
The expression of autophagic influx in MJD cells after 3-day FIR treatment. (**a**) selective autophagy-related markers (Beclin-1 and microtubule-associated protein light chain 3-II, LC3-II) and autophagy substrate (P62) were analyzed by western blot and (**b**) quantified in human neuroblastoma cells (SK-N-SH) overexpressing 26- (26Q, internal control, MJD26) and 78-CAG repeats (MJD78) in *ATXN3* with or with FIR treatment. The protein quantification was calculated by three-time independent analysis at least. *p < 0.05, compare to WT group; ^+^*p* < 0.05, compare to MJD26 group, ^#^p < 0.05, compare to each non-treated group of MJD cells.

To investigate if the observed FIR mediated ataxin-3-78Q clearance and improvement in mitochondrial quality of was associated with induction of autophagy, we measured expression of P62, LC3-II and Beclin-1 (Fig. 5a). Whereas the expression of P62 was strongly increased in both transgenic cell lines, only MJD26 cells displayed increased LC3-II levels compared with WT cells. MJD78 cells showed significant decrease in Beclin-1 and LC3-II compared to the other cell types. FIR exposure increased the expression of Beclin-1 and LC3-II in the MJD78 cells, and this was accompanied by a decrease in P62 expression (Fig. 5a,b). In contrast, exposure to FIR did not dramatically affect expression of these proteins in MJD26 cells apart from a mild increase of LC3-II (Fig. 5a,b). These results suggest that ataxin-3-78Q induced cellular stress seems to be linked to low levels of autophagy, and that FIR serves as a trigger of autophagy that counteract polyQ protein accumulation and cell damage but not for cells that carried ataxin-3-26Q which had an original high expression of autophagy marker LC3-II.

## Discussion

The presented work demonstrated that FIR treatment individually rescued cells from pathological and non-pathological mechanisms involved in MJD78 and MJD26 cells, by preventing mutant PolyQ protein accumulation in MJD78 cells and protecting mitochondrial function in both cells. The data also suggested that FIR triggered autophagy as a major rescue mechanism that did not seem to involve ROS scavenging.

FIR therapy has been used in treatment of disease such as diabetes, cardiovascular disease and chronic kidney disease. The cellular mechanisms involved remain largely unknown, but seem to involve effects on the microenvironment (e.g. blood flow), as well as intracellular factors^21^. For instance, FIR has been reported to regulate Akt-signaling and nitric oxide (NO) production in vascular endothelial cells pathway after application of FIR on cell cultures^22^ FIR is, however, likely to mediate cellular mechanisms similar to NIR, which has been more extensively as a possible therapeutic strategy in neurodegenerative diseases. NIR has been found to target photo-acceptor such as ETC complex IV^23^, which may explain the resultant increase in the activity of the complex^24^. The beneficial effects on neurons might be partly attributed to increased ATP production and protection against oxidative stress^25^; however, stimulation of mitochondrial biogenesis and anti-apoptotic/pro-survival pathways has also been reported^26^,^27^. Moreover, a recent study showed increased levels of Akt, pAkt and Bcl-2 protein levels after NIR treatment during neurotoxicity and suggests that Akt-mediated pro-survival signals might be related to the rescuing effects if NIR^28^, as well as in FIR^22^,^29^. Compared to NIR, the penetrability and heat production of FIR is small, and limited penetrability is an obstacle if one intends to treat conditions of the central nervous system where external irradiation by FIR may not be sufficient to reach the affected tissues in the brain. There are, however, potential strategies in utilizing FIR emitting materials as implants to administer such therapy as a continuous and long-term treatment^30^.

Stimulation of autophagy can inhibit SCA3 disease progression in mice models^31^. In our study ataxin-3-78Q clearance and increased viability in FIR treated cells was associated with up-regulation of Beclin-1 and LC3-II, which is an important regulator of both autophagy and apoptosis^32^. Overexpression of Beclin-1 has been found to alleviate toxic effects of mutant ataxin-3 aggregation by inducing autophagy^33^ in a neuroblastoma cell line; as well as preventing motor deficits in mouse models of SCA3^11^. On the other hand, level of P62 have also been found in increased levels in both the brains and fibroblasts of SCA3 patients and in the brains of transgenic mice with mutated ataxin-3^33^. Thus, the observed reduction in P62 level, together with restoration in LC3-II, Beclin-1 and compensational increase of *ATXN3* gene expression, suggests that autophagy is involved in the protective effects seen FIR exposed cells. Accordingly, induction of autophagy would provide a mechanism to enable clearance of ataxin-3-78Q protein. Interestingly, FIR induced decreases not only in pathogenic ataxin-3-78Q but also for the endogenous ataxin-3. Therefore, we do not rule out other possible mechanisms such as autophagic turnover of protein and organelle^34^ involved in the effects of FIR. Although the autophagy activation was not obviously observed in FIR-treated MJD26 cells, we suggested that it could be related with the overexpressing 26-CAG repeats in *ATXN3*-induced high protein level of LC3-II accompanying normal expression of Beclin-1, which balance the degradation of extensive P62 in autophagic flux. FIR exposure supported cell viability of MJD26 under rotenone-induced cell stress and the mechanism was related with mitochondrial functional support not dependent on autophagy activation. On the other hand, decreased in ataxin-3 protein in contrary to its mRNA level in FIR-treated MJD78 cells seems to reflect the possibility of FIR-mediated reducing of ataxin-3 protein’s half-life due to an increase rate of autophagic degradation. The short half-life protein is susceptible to RNA fluctuations caused by stimulations^35^ because the protein numbers control the negative feedback mechanism of translational and transcription rate^36^. Any decrease in protein numbers is compensated by the increases of the transcriptional and translational rates^36^,^37^. Herein, we did not have enough evidence to clarify the complex regulation of mRNA-protein expression under FIR treatment because its relationship according to key processes in determination of protein abundance including the transcription, mRNA decay, translation, and protein degradation^38^. Thus, it can be understood why a gene’s mRNA level could not predict its protein level especially for some cells with improper protein turnover.

SCA3 pathology is associated with mitochondrial dysfunction^7^. The normal form of ataxin-3 has been found to regulate the activity of Parkin^39^, which is central in mitochondrial quality control^40^,^41^. Interestingly, an interaction with mutated ataxin-3 was found to compromise the stability of Parkin, which suggests a resultant impairment of mitochondrial quality control^42^. Such a mechanism is compatible with our findings that ataxin-3-78Q expression leads to mitochondrial dysfunction, and that FIR treatment rescued the cells by protecting against these effect. Furthermore, we found the mitochondria in MJD78 cells to be more fragmented than healthy cells, which is a phenomenon often associated with cellular stress, respiratory dysfunction, poor mitochondrial quality, impairment of autophagy, and cell death^43^. This could therefore explain why FIR can improve mitochondrial function in MJD78 cells and why this was not accompanied by an increase of mitochondrial biogenesis. The specific increase in mitochondrial ROS production in FIR-exposed MJD78 cells, without a significant effect on total ROS and superoxide, may result from the increased rates of mitochondrial respiration, but this did not seem to correlate with toxicity. Interestingly, this would be in accordance with recent findings that local mitochondrial ROS production may actually contribute to mitochondrial quality control by engaging autophagy^44^,^45^.

In summary, our study provides evidence for beneficial effects of FIR exposure in a cell model of SCA3. Apparently, FIR therapy protects vital functions in the cells by facilitating clearance of the mutated protein, and support ATP production via mitochondrial respiration. Further investigation will need to clarify the underlying mechanisms including proteasome-ubiquitin protein degradation and mitophagy in more detail, and evaluate the therapeutic potential of FIR treatment in neurodegenerative diseases.

## Methods

### Cell culture

Wild-type human neuroblastoma cell line (SK-N-SH) was provided by Prof. Mingli Hsieh (Department of Life Science, Tunghai University, Taiwan). SK-N-SH cells were grown in Dulbecco’s modified Eagle’s medium (DMEM) (high glucose, DMEM-HG) (GIBCO/Invitrogen, Carlsbad, CA, USA) containing 10% heat-inactivated fetal bovine serum (FBS, GIBCO), 1% L-glutamine (GIBCO), 1% NEAA (GIBCO) and 1% penicillin/streptomycin (GIBCO). The medium was changed every 2 days in each and different type of cells was sub-cultivated weekly at a ratio of 1:2 and maintained at 37 °C in a humidified atmosphere with 5% CO_2_.

### Plasmids and transfection

SK-N-SH cells expressed 26 (MJD26) or 78 CAG repeats (MJD78) in ataxin-3 gene were established follow the previous study^46^. Full-length ataxin-3 constructs containing 26 or 78 CAG repeats in pEGFP-N1 vector (a kind gift from prof. Mingli Hsieh at Tunghai University) was transfected using a BTX ECM 830 electroporator (Harvard Apparatus, Holliston, MA). Transfected cells were selected in a culture medium supplemented with 0.4 mg/ml G418 (Amresco, Solon, OH, USA) to get stable clones. Selected G418-resistant cells were subcloned and maintained in the same medium for 2 months until each cell line contained homogenous transfected cells.

### FIR exposure

For irradiation, the medium was replaced and the lid removed. The cells were exposed to radiation from a WS TY301 FIR emitter (Far IR Medical Technology, Taipei, Taiwan). This FIR emitter generated electromagnetic wavelengths and energy in the range of 3-1000 μm and 3–25 μm with peak value among 5–7 μm, respectively. The radiator was set at a height of 20 cm above the bottom of the culture plates for each time exposure of 10 min, a 3-day course of three-times daily treatment, in a Live Cell system culture chamber (Pathology Devices, Westminster, MD, USA) with 37 °C and 5% CO_2_. Simultaneously, a negative control covered with aluminum foil was also set at same condition. After 3-day course of daily treatment, cells were recovered overnight and ready to analyze.

### RNA extraction and quantitative real-time RT-PCR analysis

Total RNA from cells was isolated using the TRIzol reagent (Invitrogen). Total RNA was further purified with NucleoSpin RNA II Kit (Macherey-Nagel, Düren, Germany) and reverse-transcribed using the Transcriptor First Strand cDNA Synthesis kit (Roche Applied Science, Indianapolis, USA). The expressions of mRNA were determined by quantitative analysis of real-time RT–PCR using SYBR Green PCR Master Mix (Roche Applied Science) and an ABI Prism 7300 system (Applied Biosystems). The primers targeted at non CAG-repeated region of *ATXN3* (Forward: 5′-AAGAGACGAGAAGCCTAC-3′ and Reverse: 5′-TTCACTCATAGCATCACCTA-3′) and β-actin (Forward: 5′-ATCGTGCGTGACATTAAGAGAAG-3′ and Reverse: 5′-AGGAAGGAAGGCTGGAAGAGTG-3′) used for RT-PCR were applied. PCR was performed as follows: 1 cycle of hot start at 95 °C for 10 minutes and 40 cycles of 30-second denaturation at 95 °C, annealing and extension at 60 °C for 90 seconds. The mRNA expressions normalized to β-actin respectively were presented as relative expression levels.

### Western blot analysis

Treated cells recovered overnight were washed with PBS and collected into RIPA buffer (50 mM Tris-cl pH 7.4, 150 mM NaCl, 1% NP40, 0.25% Na-deoxycholate, 1 mM PMSF) (Pierce Biotechnology, Inc. Rockford, USA) contained protein inhibitors cocktail (Sigma-Aldrich, St Louis, MO, USA), incubated on ice for 20 minutes and homogenized. The extract was then spun at 14,000 × g for 10 minutes at 4 °C and the supernatant was analyzed by BCA assay (Pierce, Rockford, IL, USA). Aliquots of whole-cell lysates with 25 μg of protein were fractioned and separated electrophoretically on a 12% SDS-polyacrylamide gel (Bio-Rad Laboratories, Richmond, CA) and blotted onto a piece of polyvinylidene difluoride membrane (Amersham Biosciences, Buckinghamshire, UK). Non-specific binding was blocked with 5% skim milk in Tris-buffered saline with Tween 20 (Sigma-Aldrich). The membrane was blotted with primary antibodies that included Ataxin 3 (1:1000 dilution, Abcam, Cambridge, UK), P62 (1:1000 dilution, Abcam), LC3 (1:1000 dilution, Novus Biologicals, Littleton, CO, USA), Beclin 1 (1:1000 dilution, Abcam), OPA1 (1:500 dilution, BD Biosciences Pharmingen, San Diego, CA, USA), MFN2 (1:500 dilution, Sigma-Aldrich), Fis1 (1:500 dilution, Axxora, San Diego, CA), Drp1 (1:500 dilution, Abcam), Tom20 (1:1000 dilution, Santa Cruz, California, USA), Tim23 (1:1000 dilution, Santa Cruz) and GAPDH (1:1000 dilution, Millipore, Billerica, CA, USA). After incubation with a specific horseradish peroxidase-conjugated secondary antibody (Jackson Immunoresearch Lab, West Grove, PA, USA), the protein intensity was determined by an enhanced chemiluminescence reagent (Immobilon Western, Millipore, Billerica, CA, USA) and images were captured with the Fusion FX7 system (Vilber Lourmat, Marne-la-Vallée, France).

### Seahorse XF24 Extracellular Flux analyzer

Thirty thousand of treated cells per well were seeded in the XF24 microplate with normal condition medium for 16–18 hours before mitochondrial function assay. According to manufacturer’s recommended protocol of seahorse XF24 extracelluar flux analyzer, cell medium was replaced by the conditional medium (culture medium without FBS and sodium bicarbonate) and incubated at the incubator without supplied CO_2_ for one hour before completion of probe cartridge calibration. Basal oxygen consumption rate (OCAR) was measured in the Seahorse XF24 Flux analyzer. Measurements were performed after injection of three compounds affecting bioenergetics: 1 μM oligomycin (Sigma, St Louis, MO, USA), 0.5 μM carbonyl cyanide 4-(trifluoromethoxy)phenylhydrazone (FCCP) (Sigma) and 2 μM Rotenone (Sigma). Upon completion of the Seahorse XF24 Flux analysis, cells were lysed to calculate the protein concentration using BCA assay (Pierce). The result was normalized with the protein OD value of corresponding well.

### Analysis of mitochondrial morphology

For visualization of mitochondria, cells after 3-day treatment were seeding at a chamber slide (μ-Slide 8 well, Ibidi GmbH, Munich, Germany) and stained with Mitotracker Green (250 nM) (Invitrogen, Carlsbad, CA, USA) at incubator for 40 min. Staining cells were removed remained dye and mounted on a perfusion chamber in culture media and imaged at 37 °C using an Olympus FluoView FV 1200 Confocal Microscope (Olympus, Tokyo, Japan). The mitochondrial morphology was quantified by random calculation of mitochondrial diameter using the CellSens Dimension Desktop version 1.3 (Olympus Corporation) software. Considering the error of automatic analysis caused by mitochondrial aggregation, diameter greater than 15 μm was not assessed.

Analysis of mitochondrial morphological subtyping was executed by using an automatic classification system according to Peng *et al*. study^47^. After semi-automatic segmentation of cell micrograph from confocal microscopy, mitochondrial extraction, involved segmentation each image into mitochondria and background, and classification of six distinct and meaningful mitochondria subtypes (small globe, swollen globe, straight tubule, twisting tubule, branch tubule and loop) have been done by an automatic classification software. Color-encoding labeling of morphological subtypes were automatically generated. The micrograph from three independent areas in each group was analyzed, there were about 250–350 mitochondrial subjects from about 4–6 cells in each image was calculated.

### Mitochondrial copy number

An aliquot of 20 ng of DNA was subjected to quantitative PCR by Applied Biosystems QPCR 7300 instrument (Foster City, CA, USA) using LightCycler-FastStar DNA Master SYBR Green I kit (Roche Applied Sciences) referred to^48^. The primers were as following: for mtDNA-ND1: Forward 5′-AACA TACCCATGGCCAACCT-3′; Reverse 5′-AGCGAAGGGTTGTAGTAGCCC-3′. For β-globin: Forward 5′-GAAGAGCCAAGGACAGGTAC-3′; Reverse 5′-CAACTTCATCCACGTTCACC-3′. The relative mtDNA copy number was measured by normalization of the crossing points in quantitative PCR curves between ND1 and β-globin genes using the RelQuant software (Roche Applied Sciences).

### Cell viability

Cell viability and sensitivity of rotenone (100 nM)-induced cell death was assessed by retention of propidium iodide (PI, Invitrogen) uptake using a flow cytometry (FC500, Beckman Coulter). Treated cells with or without 24 h induction of 100 nM Rotenone were rinsed carefully with PBS and incubated with 10 μg/ml PI in PBS at 37 °C for 15 min. cell survival was assessed by deducting the percentage of dead (PI-positive cells) to the sum of total cells.

### Oxidative stress

Dihydrofluorescein diacetate (10 μg/ml, DCFH-DA, Invitrogen) was used to stain for total cellular hydrogen peroxide, dihydroethidium (20 μM, Invitrogen) for cellular superoxide, and MitoSox Red (5 μM, Invitrogen) for mitochondrial superoxide. For FACS analysis, 1 × 10^6^ cells were stained in PBS with the indicated fluorescent dye for 25 min, washed, and resuspended in 800 μl PBS with 1% FCS. Individual cellular fluorescence signals were then analyzed using a flow cytometry.

### Immunofluorescent staining and confocal microscopy

Cells were fixed by treating with 4% paraformaldehyde in PBS (pH = 7.4) for 15 min. After twice wash with PBS, 0.5% Triton X-100 in PBS (pH = 7.4) was applied to penetrate cell membrane for 5 min. To reduce the nonspecific antibody binding, the cells were treated with 5% normal bovine serum in PBS for 15 min then incubated overnight at 4 °C with monoclonal antibodies overnight against rabbit phosphate Drp1 (Ser616) antibody (1:400 dilution, Cell Signaling Technology, Danvers, Massachusetts, USA). After more than three time wash with PBS, cells were then incubated for 1-h with goat anti-rabbit secondary antibodies conjugated to DyLight 488 (1:500 dilution, Jackson ImmunoResearch, West Grove, Pennsylvania, USA). After nuclear counterstain with 10 μg/mL Hoechst 33342 (Invitrogen Molecular Probes) for 20 min, cells were then mounted onto glass slides and cover-slipped with Histochoice mounting media (Amresco, Solon, OH, USA). Confocal microscopy was performed using an inverted laserscanning microscope (FV1200 confocal microscopy system (Olympus). The images of fluorescence intensity were analyzed using FLUOVIEW software.

### Statistical analysis

Experiments presented were repeated at least three times with triplicate samples. The data are presented as the mean ± SD. Comparison of two experimental conditions was evaluated using the paired Student’s t-test. A difference with *p* < 0.05 was considered to be statistically significant.

## Additional Information

**How to cite this article**: Chang, J.-C. *et al*. Far-infrared radiation protects viability in a cell model of Spinocerebellar Ataxia by preventing polyQ protein accumulation and improving mitochondrial function. *Sci. Rep*. **6**, 30436; doi: 10.1038/srep30436 (2016).

## Supplementary Material

Supplementary Information
