# Supplementary material for: Far-infrared radiation protects viability in a cell model of Spinocerebellar Ataxia by preventing polyQ protein accumulation and improving mitochondrial function
Source: Sci Rep. 2016 Jul 29;6:30436. doi: 10.1038/srep30436 (PMC4965738; doi:10.1038/srep30436)
Supplement: Supplementary Information [file srep30436-s1.doc]

**Far-infrared radiation protects viability in a cell model of Spinocerebellar Ataxia by preventing polyQ protein accumulation and improving mitochondrial function**

Jui-Chih Chang^a+^, Shey-Lin Wu^b+^, Fredrik Hoel^e^, Yu-Shan Cheng^b^, Ko-Hung Liu^b^, Mingli Hsieh^f^, August Hoel^e^, Karl Johan Tronstad^e^, Kuo-Chia Yan^c^, Ching-Liang Hsieh^gi^, Wei-Yong Lin^hi^, Shou-Jen Kou^d*^, Shih-Li Su^jk^, Chin-San Liu^a,b,i*^

^a^Vascular and Genomic Center, ^b^Department of Neurology, ^c^Department of Dermatology, ^d^Department of Surgery ,Changhua Christian Hospital, Changhua 50094, Taiwan

^e^Department of Biomedicine, University of Bergen, 5020 Bergen, Norway

^f^Department of Life Science, Tunghai University, Taichung 40704, Taiwan

^g^Department of Chinese Medicine, ^h^Departments of Medical Research, Obstetrics and Gynecology, Dermatology, and Urology, China Medical University Hospital, Taichung 40447, Taiwan

^i^School of Chinese Medicine, Graduate Institute of Integrated Medicine, Research Center for Chinese Medicine and Acupuncture, China Medical University, Taichung 40447, Taiwan

^j^Division of Endocrinology and Metabolism, Department of Internal Medicine, Changhua Christian Hospital, Changhua 50094, Taiwan

^k^Institute of Medicine, Chung Shan Medical University, Taichung 40201, Taiwan

**^+^co first-author**

**^*^ corresponding author**

Chin-San Liu, MD, PhD

E-mail: liu48111@gmail.com

Department of Neurology and Vascular and Genomic Center

Changhua Christian Hospital

135 Nanhsiao Street, Changhua 50094, Taiwan

Shou-Jen Kou, MD, PhD

E-mail: 40225@cch.org.tw

Department of Surgery

Changhua Christian Hospital

135 Nanhsiao Street, Changhua 50094, Taiwan

**Supplementary Information**

**Result**

Expressions of the respective polyQ-expanded ataxin-3 protein with a mass of 67 KDa and 73 KDa in the MJD26 and MJD78 cells as well as endogenous (Endo) ataxin-3 protein were confirmed by western blotting in different cell passage cell numbers (Fig. S1). Both of MJD26 and MJD78 expressed dramatic increases of endogenous ataxin-3 and polyQ-expanded ataxia-3 protein compared to the wild type (WT) in each late late-passage cells. We suggested that the compensational increase of wild-type ataxin-3 protein in MJD cells was in order to against polyQ accumulation-induced stress caused by long-term culture ^1^.

Figure S1: Effect of cell passage numbers on ataxin-3 protein in MJD cells. Endogenous ataxia-3 (48-kDa-protein, Endo) and polyQ-expanded ataxin-3 were examined in the early- and late-passage MJD26 (67-kDa-protein, 26Q; N=6 vs. N=20) and MJD78 cells (73-kDa-protein, 78Q; N=6 vs. N=21).


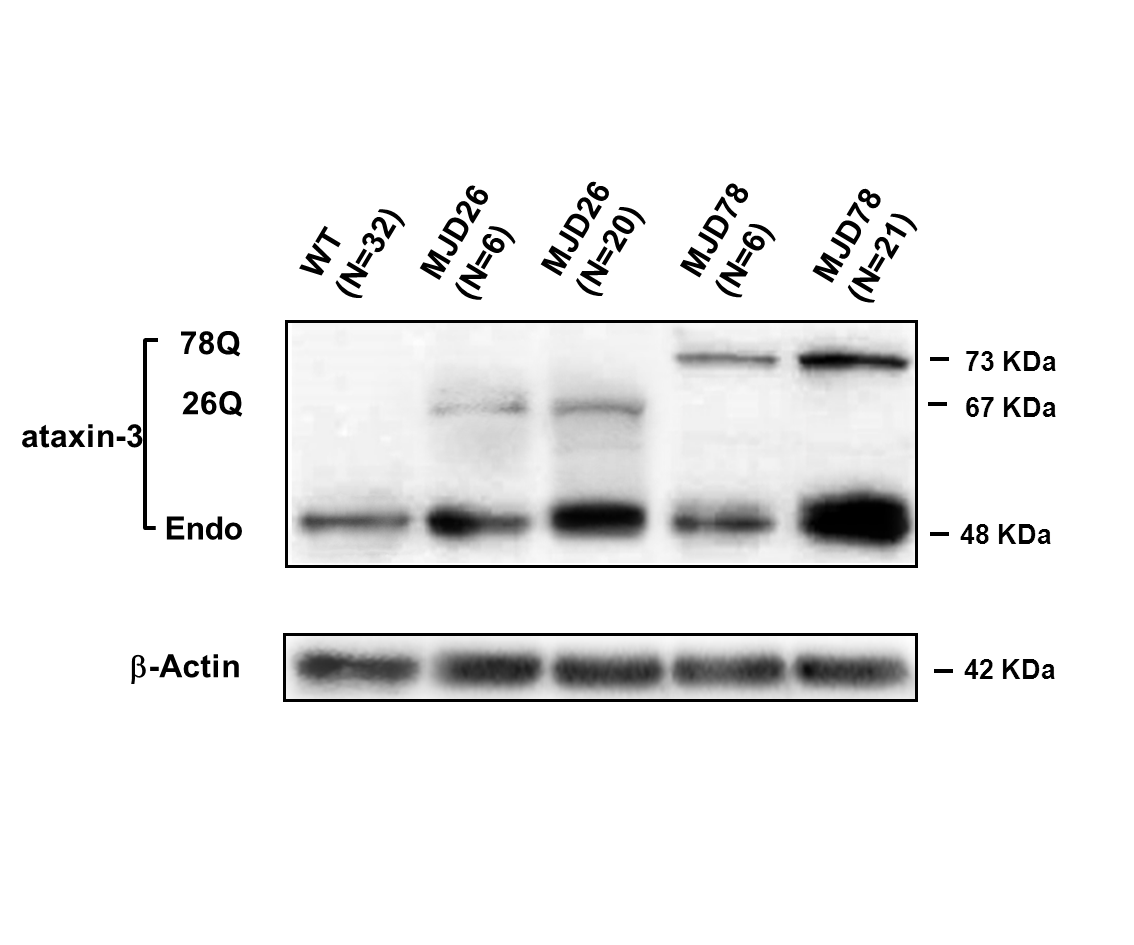


**References**

1 Reina, C. P., Nabet, B. Y., Young, P. D. & Pittman, R. N. Basal and stress-induced Hsp70 are modulated by ataxin-3*. Cell Stress Chaperon*e**s** 17, 729-742, doi:10.1007/s12192-012-0346-2 (2012).
